# A Deep Learning Approach to Automatic Tooth Caries Segmentation in Panoramic Radiographs of Children in Primary Dentition, Mixed Dentition, and Permanent Dentition

**DOI:** 10.3390/children11060690

**Published:** 2024-06-05

**Authors:** Esra Asci, Munevver Kilic, Ozer Celik, Kenan Cantekin, Hasan Basri Bircan, İbrahim Sevki Bayrakdar, Kaan Orhan

**Affiliations:** 1 Department of Pediatric Dentistry, Faculty of Dentistry, Ataturk University, Erzurum 25240, Turkey; esra_6221@hotmail.com (E.A.); hasanbasri.bircan@ogr.atauni.edu.tr (H.B.B.); 2 Department of Pediatric Dentistry, Faculty of Dentistry, Beykent University, İstanbul 34398, Turkey; 3 Department of Mathematics Computer, Faculty of Science, Eskisehir Osmangazi University, Eskisehir 26040, Turkey; ozercelik05@gmail.com; 4 Center of Research and Application for Computer Aided Diagnosis and Treatment in Health, Eskisehir Osmangazi University, Eskisehir 26040, Turkey; ibrahimsevkibayrakdar@gmail.com; 5 Department of Pediatric Dentistry, Faculty of Dentistry, Sakarya University, Sakarya 54050, Turkey; k_cantekin@hotmail.com; 6 Department of Oral and Maxillofacial Radiology, Faculty of Dentistry, Eskisehir Osmangazi University, Eskisehir 26040, Turkey; 7 Department of Oral and Maxillofacial Radiology, Faculty of Dentistry, Ankara University, Ankara 06620, Turkey; call53@yahoo.com

**Keywords:** caries, Artificial Intelligence, panoramic radiography, deep learning

## Abstract

Objectives: The purpose of this study was to evaluate the effectiveness of dental caries segmentation on the panoramic radiographs taken from children in primary dentition, mixed dentition, and permanent dentition with Artificial Intelligence (AI) models developed using the deep learning method. Methods: This study used 6075 panoramic radiographs taken from children aged between 4 and 14 to develop the AI model. The radiographs included in the study were divided into three groups: primary dentition (n: 1857), mixed dentition (n: 1406), and permanent dentition (n: 2812). The U-Net model implemented with PyTorch library was used for the segmentation of caries lesions. A confusion matrix was used to evaluate model performance. Results: In the primary dentition group, the sensitivity, precision, and F1 scores calculated using the confusion matrix were found to be 0.8525, 0.9128, and 0.8816, respectively. In the mixed dentition group, the sensitivity, precision, and F1 scores calculated using the confusion matrix were found to be 0.7377, 0.9192, and 0.8185, respectively. In the permanent dentition group, the sensitivity, precision, and F1 scores calculated using the confusion matrix were found to be 0.8271, 0.9125, and 0.8677, respectively. In the total group including primary, mixed, and permanent dentition, the sensitivity, precision, and F1 scores calculated using the confusion matrix were 0.8269, 0.9123, and 0.8675, respectively. Conclusions: Deep learning-based AI models are promising tools for the detection and diagnosis of caries in panoramic radiographs taken from children with different dentition.

## 1. Introduction

Dental caries is a common chronic infectious condition that affects many children, young adult and adult individuals worldwide [1,2]. Although dental caries usually progress slowly, in the absence of appropriate early intervention, they can become a serious health issue causing pain, infection, and tooth loss [3]. In clinical dentistry, caries detection involves the determination of treatment, the assessment of the level of caries risk, and the application of preventive methods, and is very important in guiding clinical planning [4]. Successful treatment requires timely and accurate diagnosis. Various diagnostic methods are used, including digital subtraction radiography (DSR), optical coherence tomography (OCT), electrical conductivity measurement (ECM), ultrasonic imaging, fibre-optic transillumination (FOTI), laser fluorescence, and quantitative light-induced fluorescence (QLF) [5]. The interpretation of the images acquired by these methods is limited by inter-rater disagreement, and no single method alone can diagnose caries on the entire tooth surface. The ideal method for diagnosing dental caries has not yet been found. In this quest, interest in caries detection with computer-aided image analysis is increasing. 

Panoramic radiography has found favour as an extraoral method, and interest has increased owing to its low radiation dose, lower time necessity, ease of application, and greater patient comfort [6]. However, extraoral imaging methods are associated with the distortion and magnification of images [7]. Panoramic radiography singly is inferior to bitewing radiography in the diagnosis of caries [6,8]. However, with the technological developments in panoramic radiography devices, it is now able to compete with intraoral imaging in the diagnosis of caries in panoramic radiographs [9]. Intraoral radiography necessitates more patient cooperation in comparison with extraoral techniques. Hence, paediatric and disabled patients could benefit greatly from an extraoral imaging system.

Artificial Intelligence (AI) methodologies, specifically deep learning-based convolutional neural networks (CNN), have shown good performance in computer communications, including object, face, and activity tracking, recognition, three-dimensional mapping, and localisation [10]. Image processing and image recognition procedures have been applied in medical image segmentation and diagnosis. The U-Net is a convolutional network architecture used for the fast and precise segmentation of biomedical images, and the U-Net architecture has been reported to achieve successful results in medical image datasets. The U-Net architecture can run on a trained dataset with fewer images and provide precise segmentation. However, research on the application of deep CNN infrastructure and studies on caries’ diagnostic methods in dentistry has not yet reached a common conclusion [11]. This study was performed to evaluate the efficacy of an AI application developed using deep learning methods for dental caries diagnosis on panoramic radiographs of children with primary, mixed, and permanent dentition.

## 2. Materials and Methods

### 2.1. Patient Selection

This study is a retrospective study. This study was approved by Ataturk University Medical Faculty Clinical Research Ethics Committee with the decision no. 04/30. Panoramic radiographs of 6075 paediatric patients aged 5–14 years that were available in the radiology archive of Ataturk University were included in the presented study. Since this study is an archival study, consent was not obtained from the patients retrospectively. Panoramic radiographs containing any artefacts were excluded from the study dataset. Panoramic radiographs with caries lesions deep enough to be visible on the radiograph were selected due to visuality. Panoramic radiographs with orthodontic appliances, types of restorations (stainless steel crowns, space maintainer), and containing periodontal and periapical lesions were also included in the dataset. The panoramic radiographs were divided into three groups: primary dentition, mixed dentition, and permanent dentition. In addition, all radiographs were evaluated in a single category. 

### 2.2. Radiographic Data

All images used in this study were acquired at 65 kVp, 8 mA, and 16 s using the Planmeca Promax 2D Panoramic system (Planmeca, Helsinki, Finland).

### 2.3. Image Evaluation

**Figure 1:**
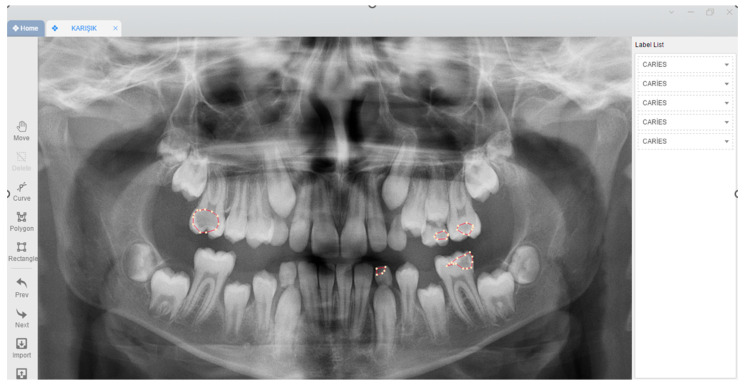
Caries labelling on panoramic images with polygonal tool using the Colabeler labelling software (MacGenius, Blaze SoftwareNewport Beach, CA, USA).

Each caries label on panoramic images was annotated with a polygonal tool by a research assistant (E.A.) with 3 years of experience and a pedodontist (M.K.) with 10 years of experience using the Colabeler labeling software (MacGenius, Blaze Software, Newport Beach, CA, USA) (Figure 1). In the study, all panoramic radiographs were evaluated by two specialists separately, initially. Then, these radiographs were evaluated together again by two specialists and the last common decision was taken. All panoramic radiographs in which the specialists did not agree were excluded from the dataset to minimize the possibility of missing caries lesions on panoramic radiographs. The bounding box method (rectangular boxes) was used for caries detection. It determines the location of decayed teeth.

### 2.4. Deep Convolutional Neural Network Architecture

U-Net architecture was used as the Deep Convolutional Neural Network Architecture. The U-Net architecture is used for semantic segmentation tasks. Our encoder–decoder type consisted of four block levels, including two convolutional layers, with a max-pooling layer in the encoding part and up convolutional layers in the decoding part. Each block had 32, 64, 128, or 256 convolutional filters. In addition to the bottleneck, the layer contained 512 convolutional filters [12].

### 2.5. Model Pipeline

The Python open-source programming language (v.3.6.1; Python Software Foundation, Wilmington, DE, USA) and the PyTorch library (version 1.4.0) were used for model development. Model training was conducted on a computer equipped with 16 GB RAM and an NVIDIA GeForce GTX 1060Ti graphics card. Prior to training, all panoramic radiographs were resized from 2943 × 1435 to 1024 × 512 pixels.

**Figure 2:**
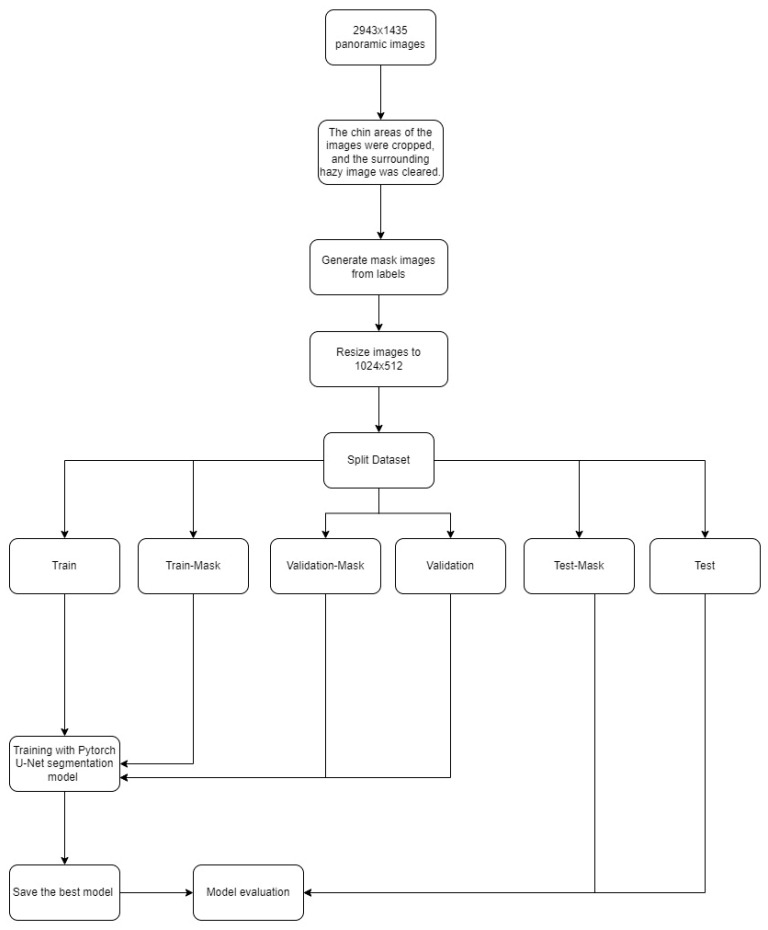
Diagram of the development stages of the AI models for primary dentition, mixed dentition, and permanent dentition.

Primary dentition: The dataset consisted of 1857 images, with 1497 images (12,203 labels) in the training set, 180 images (1276 labels) in the validation set, and 180 images (1551 labels) in the testing set. Labelling was carried out more than once on a tooth. Caries in separate areas were evaluated separately. The panoramic radiographs were randomly distributed. Two hundred epochs were trained with the PyTorch U-Net model; epoch 149 showed the best performance and was therefore used in the model (Figure 2). 

Mixed dentition: The dataset consisted of 1406 images, with 1126 images (6252 labels) in the training set, 140 images (674 labels) in the validation set, and 140 images (760 labels) in the testing set. The images were randomly distributed. Two hundred epochs were trained with the PyTorch U-Net model; epoch 176 showed the best performance and was therefore used in the model (Figure 2). 

Permanent dentition: The dataset consisted of 2812 images, with 2242 images (10,152 labels) in the training set, 285 images (1130 labels) in the validation set, and 285 images (1102 labels) in the testing set. The images were randomly distributed. Two hundred epochs were trained with the PyTorch U-Net model; epoch 155 showed the best performance and was therefore used in the model (Figure 2). 

### 2.6. Total (Primary Dentition + Mixed Dentition + Permanent Dentition)

The dataset consisted of 4875 images, with 2242 images (28,014 labels) in the training set, 600 images (3567 labels) in the validation set, and 600 images (3463 labels) in the testing set. The images were randomly distributed. One hundred epochs were trained with the PyTorch U-Net model; epoch 75 showed the best performance and was therefore used in the model (Figure 2). 

### 2.7. Statistical Analysis

A confusion matrix was used to assess the model performance. The metrics used in this matrix were as follows: TP (true positive), the rate of positive cases correctly predicted; FN (false negative), the ratio of negative values incorrectly classified as positive; and FP (false positive), the rate of negative cases correctly classified. The metrics used to evaluate the model success were as follows: precision, a measure of how many correct predictions were made out of all classes (TP/TP + FP); sensitivity, an indicator of the model efficacy in predicting the positive class label from the inputs (TP/TP + FN); and F1 score, the harmonic mean of precision and sensitivity.

## 3. Results

The success of the AI model in caries diagnosis was evaluated in each of the groups.

**Figure 3:**
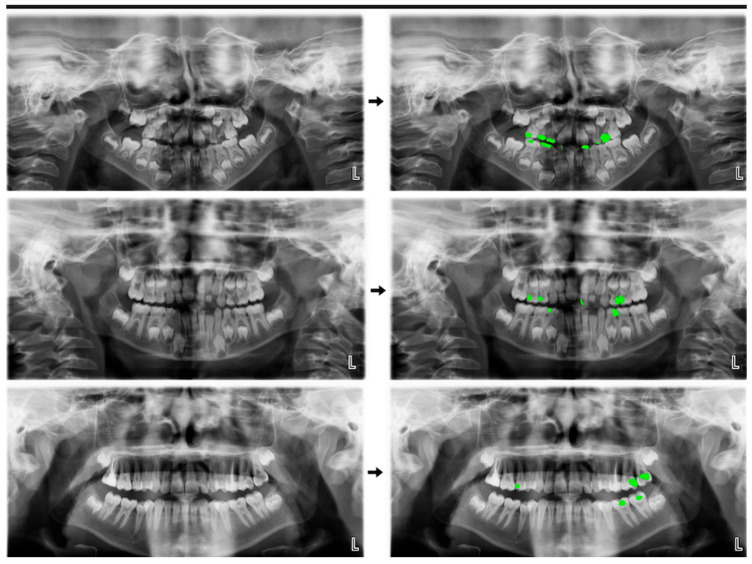
Caries segmentation using an AI model on panoramic radiographs of children with primary dentition, mixed dentition, and permanent dentition.

Primary Dentition: Among the 1276 caries labels on 180 images in the testing set, the AI system evaluated 1006 as TP, 96 as FP, and 174 as FN (Figure 3). The sensitivity, precision, and F1 score calculated using the confusion matrix were 0.8525, 0.9128, and 0.8816, respectively (Table 1). 

Mixed Dentition: Among the 674 caries labels on 140 images in the testing set, the AI system evaluated 467 as TP, 41 as FP, and 166 as FN (Figure 3). The sensitivity, precision, and F1 score calculated using the confusion matrix were 0.7377, 0.9192, and 0.8185, respectively (Table 1).

Permanent Dentition: Among the 1130 caries labels on 285 images in the testing set, the AI system evaluated 866 as TP, 83 as FP, and 181 as FN (Figure 3). The sensitivity, precision, and F1 score calculated using the confusion matrix were 0.8271, 0.9125, and 0.8677, respectively (Table 1). 

**Figure 4:**
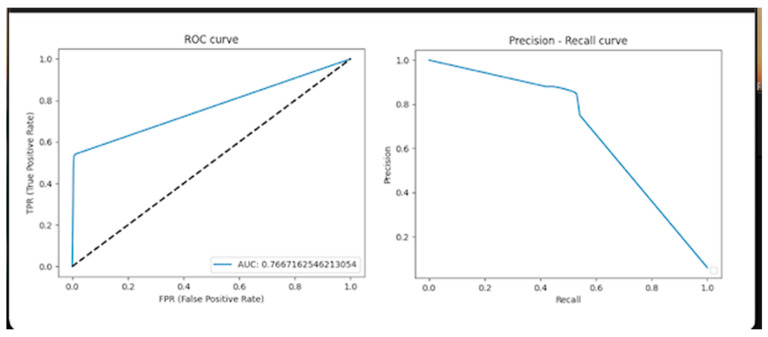
ROC and precision–recall curve for total caries segmentation model including primary dentition, mixed dentition, and permanent dentition.

Total (Primary dentition + Mixed dentition + Permanent dentition): Among the 3463 caries labels on 600 images in the testing set, the AI system evaluated 2653 as TP, 255 as FP, and 555 as FN (Figure 3). The sensitivity, precision, and F1 score calculated using the confusion matrix were 0.8269, 0.9123, and 0.8675, respectively (Table 1). The area under curve (AUC) value was found to be 0.76 (Figure 4).

## 4. Discussion

If dental caries is not detected correctly and early, the lesion may gradually extend into the dentin, enamel, and even the tooth pulp, resulting in severe pain and consequently the loss of dental function. Artificial intelligence-based systems are often used in dentistry for the design of automated software to facilitate diagnosis and data management [13]. These are often clinical decision support systems that help and guide professionals to make better decisions. These systems have been used to improve the diagnosis, treatment planning, and prediction of prognosis [14]. This study was performed to examine the success of an artificial intelligence application developed using deep learning in the diagnosis of dental caries on panoramic radiographs of primary, mixed, and permanent dentition.

Various diagnostic methods are being developed and improved to overcome clinical and radiographic diagnostic limitations [5]. The techniques now used in clinical settings include digital subtraction radiography (DSR), optical coherence tomography (OCT), laser fluorescence, electrical conductivity measurement (ECM), ultrasonic imaging methods, digital imaging fibre-optic transillumination (DIFOTI), and quantitative light-induced fluorescence (QLF) [15,16]. Takeshita et al. demonstrated that DSR had high sensitivity and specificity in diagnosing interproximal caries [17]. In this method, however, it is important to acquire standard and good-quality radiographs via film holders. The use of artificial intelligence has great potential for eliminating errors that may not be noticed or may be overlooked by the human eye [18]. Laitala et al. evaluated the validity of the DIFOTI method by comparison with visual inspection and bitewing radiography, but found that the method had low sensitivity and was subjective [19]. Subjectivity in a method prevents the application of a standard procedure for that method. In the present study, we reduced subjectivity through an artificial intelligence system developed using deep learning on standardised panoramic radiographs. DIAGNOdent Pen, a laser fluorescence (LF) device with no X-ray exposure, is used for caries detection [20]. However, it has been reported that LF-derived scores are weakly associated with caries histology [21]. In addition, this LF device can produce FP responses as it is affected by a discolouration of the tooth surface and dental plaque [22,23]. Radiographs reflect structural changes in the tooth without being affected by discolouration or plaque. This feature can increase the reliability of the results achieved on panoramic images. The study by Mansour et al. using LF and OCT diagnostic methods established that LF could detect caries at restoration margins, but not underneath restorations [24]. These differences among caries detection methods suggest that the reliability of a method alone is not sufficient [25]. 

Panoramic radiography is one of the most preferred methods for patient evaluation in a routine paediatric examination, as it is well tolerated by children and gives an image area that covers the whole mouth [26]. Panoramic radiography can increase the accuracy and reliability of caries diagnosis through artificial intelligence applications compared to bitewings as these radiographs provide the data needed through deep learning methods as a whole.

A review by Schwendicke et al. reported that classification and segmentation could be performed using CNNs on periapical, bitewing, CBCT, and panoramic radiographs for the detection of caries and anatomical structures, and that the most frequently used method was panoramic radiography [27]. Although radiographic methods such as bitewing radiography are commonly used in caries detection, these methods only detect caries in a certain area and are therefore insufficient for an assessment of caries for all teeth, as is the case with panoramic radiography [7]. Vinayahalingam et al. [28] obtained demonstrable accuracy in their study on the classification of caries in third molars on panoramic radiographs using deep learning. The present study evaluated caries detection via the application of artificial intelligence in panoramic radiography, providing information about all teeth for caries risk assessment. 

In the area of machine learning, and especially the problem of statistical categorisation, the confusion matrix, also known as an error matrix, is a specific table layout that allows the visualisation of the performance of an algorithm by summarising predicted and actual instances [29]. Yasa et al. used a confusion matrix in their study, and evaluated the performance of a model using TP, FP, and FN, but not true negative (TN), as metrics [30]. The present study also employed the confusion matrix, using TP, FP, and FN to evaluate the performance for caries detection. TN could not to counted, because the presented AI model was developed to segment caries lesions. Only decayed teeth were labelled on panoramic images. Healthy teeth were not labelled in any way. In future studies, AI models should be developed to classify teeth that have caries or do not have caries. Cascade networks should be developed to classify teeth and segment caries lesions. U-Net is a convolutional network architecture used for the fast and precise segmentation of biomedical images [31]. Nishitani et al. reported that the U-Net deep learning algorithm is suitable for the segmentation of teeth on panoramic images [32]. Therefore, in the present study, the U-Net model, which has a high rate of success in medical image segmentation, was preferred for segmentation in the deep learning model.

Major deep learning libraries consist of layer-based frameworks, such as Caffe, and graph-based frameworks, such as PyTorch, TensorFlow, and MXNet [33]. Torch is an open-source library developed to support deep learning and machine learning [34]. This library is used frequently in image processing [35] and has been shown to simplify complex operations [36]. Therefore, the present study used the Python open-source programming language and PyTorch deep learning library, which were shown to be successful in the development of artificial intelligence models.

There are studies in the literature in which AI is used in the detection of dental caries. However, it is necessary to increase the number of these studies in order to reach a common conclusion. Lee et al. reported that dental caries could be detected with deep learning-based CNN applications on 3000 periapical images [37]. They stated that the diagnostic accuracy was 82.0%, sensitivity 81.0%, and specificity 83.0% in premolars and molars. Schwendicke et al. used DIAGNOcam and detected caries on 217 images with deep CNN [38]. Devito et al. applied a multilayer artificial neural network for proximal caries diagnosis on bitewing radiographs of 160 extracted teeth [39]. The present study used 6057 panoramic images. This high number of images in our dataset increased the reliability of our results compared to previous studies. Zhang et al.’s study was conducted on 100 panoramic images of children aged 2–6. Since it included a smaller sample and age range than this study, its results were found to be quite successful. This shows that such studies are promising [40].

In the present study, the sensitivity, precision, and F1 score were high for primary and permanent dentition, while these scores were lower for mixed dentition. High scores for permanent and primary dentition may have resulted from a clearer reading of images due to the uniform dentition in permanent dentition and the smaller size of the permanent tooth germs in primary dentition compared to the germs in mixed dentition. In mixed dentition, the development of permanent tooth germs and root resorption in primary teeth may have adversely affected the image clarity. This may explain the higher sensitivity rate for primary and permanent dentition than for mixed dentition.

This study had some limitations. The application of a method in clinical procedures requires achieving results of ≥90% [41]. Our AI method needs to be improved to achieve such results. In addition, our findings were not compared with different radiographic caries detection methods. Therefore, the use of more cases to train deep learning-based CNN systems as well as more advanced algorithms will increase the success of caries detection on panoramic radiographs and ensure a place for these systems in routine clinical practice. Because of the lack of comparisons in AI applied in dentistry, comparative studies in the latter are required. In the presented study, the cascade network was not developed. To remove the limitation, cascade AI networks should be developed to classify teeth and segment caries lesions in the future studies. At the same time, a limitation of this study is that the sample size is limited to the number of panoramic images in the archive. In addition, histological confirmations of caries and further extensions of labelled data are required, to tide over the model’s limits in the presented study. Again, comparing this study with a clinical caries detection method may provide clearer results.

## 5. Conclusions

The deep learning-based artificial intelligence algorithm reported here showed an average performance in detecting dental caries on panoramic radiographs. Prospective studies should focus on caries staging. The promising results of this study on the use of artificial intelligence to interpret dental radiographic images will encourage further studies of this issue.

### Main Points

This study sheds light on the use of artificial intelligence, which is a current topic, in dentistry. It is also one of the first studies on children’s OPTs. It gives the results of a large number of OPT scans as a contribution to the literature. In this study, the dental structure of the children was evaluated as a whole as well as separately (permanent dentition, mixed dentition and primary dentition). There were also promising results in the use of artificial intelligence in dentistry. This study, in which panoramic films were evaluated, can minimize the problems that will occur during the examination in paediatric dentistry through artificial intelligence.

## Figures and Tables

**Table 1:** Estimated caries segmentation performance measurements of the AI model on panoramic radiographs of children with primary dentition, mixed dentition, and permanent dentition using confusion matrix in primary dentition, mixed dentition, and permanent dentition.

Metrics and Measurements	Primary Dentition	Mixed Dentition	Permanent Dentition	Total (Primary + Mixed + Permanent)
True positive (TP)	1006	467	866	2653
False positive (FP)	96	41	83	255
False negative (FN)	174	166	181	555
Sensitivity	0.8525	0.7377	0.8271	0.8269
Precision	0.9128	0.9192	0.9125	0.9123
F1 score	0.8816	0.8185	0.8677	0.8675

Note: Two professional editors, both native speakers of English. For a certificate, please see http://www.textcheck.com/certificate/83DGHG, accessed on 19 May 2024.

## Data Availability

The original contributions presented in the study are included in the article, further inquiries can be directed to the corresponding author.
